# P036: In-hospital epidemics of seasonal influenza a/h3n2 in a geriatric facility

**DOI:** 10.1186/2047-2994-2-S1-P36

**Published:** 2013-06-20

**Authors:** L Pagani, V Sauvan, Y Thomas, A Iten, B Huttner, L Kaiser, D Pittet, S Harbarth

**Affiliations:** 1 Infection Control Program, Geneva University Hospitals, Geneva, Switzerland; 2 Laboratory of Virology, Geneva University Hospitals, Geneva, Switzerland

## Introduction

Seasonal influenza (SI) infection represents a threat for the elderly. We investigated a nosocomial outbreak of SI in the 300-bed geriatric facility of the Geneva University Hospitals.

## Methods

Based on surveillance of clinical influenza cases, a nosocomial outbreak was suspected at the geriatric hospital. Between 15 Jan and 30 Apr 2012, all suspected cases were screened for respiratory viruses through nasopharyngeal real-time reverse transcription-polymerase chain reaction (RT-PCR) and infection control procedures (droplet precautions with single room isolation whenever possible) were implemented. The first proven case was detected on Feb 3 and the last on Apr 2, 2012. Cases were defined as nosocomial when symptoms occurred after 72 h following hospital admission. Patients and healthcare workers (HCWs) vaccination status were also investigated**.** All positive viral specimens were processed and sequenced in order to track the transmission dynamics.

## Results

Of 155 suspected cases, 73 (47%) had RT-PCR-proven SI; 62/73 (85%) were nosocomial. 26/155 (17%) were positive for other respiratory viruses, and 60 (36%) proved negative. Among all the confirmed cases, four main clusters of nosocomial transmission were identified by viral sequencing. 43/73 (60%) patients were given oseltamivir treatment and only 4 (9%) were inappropriately treated (>72 h from disease onset). Of 23/73 (32%) patients who experienced clinical complications, 4 required enhanced care and 7 died. Over 90% of clinical complications were observed in wards admitting patients with more severe underlying diseases. Vaccine coverage among HCWs was low (30%; 116/379).

## Conclusion

Influenza remains a severe infection among hospitalized elderly patients. Very low HCW vaccination rates and gaps in recommended infection control procedures are likely to have contributed to the nosocomial spread of SI, together with the observed mismatch between the vaccine and late circulating H3N2 strain, and the potential weakness of the immune response to vaccination in the elderly. Improved strategies for the prevention and control of SI should be implemented for hospitalized geriatric patients.

## Disclosure of interest

None declared

